# Molecular Chaperone GRP94/GP96 in Cancers: Oncogenesis and Therapeutic Target

**DOI:** 10.3389/fonc.2021.629846

**Published:** 2021-04-09

**Authors:** Xiaofeng Duan, Stephen Iwanowycz, Soo Ngoi, Megan Hill, Qiang Zhao, Bei Liu

**Affiliations:** ^1^ Department of Microbiology & Immunology, Hollings Cancer Center, Medical University of South Carolina, Charleston, SC, United States; ^2^ Division of Hematology, Department of Internal Medicine, The Ohio State University Comprehensive Cancer Center, Columbus, OH, United States; ^3^ Department of Pediatric Oncology, Tianjin Medical University Cancer Institute and Hospital, National Clinical Research Center for Cancer, Key Laboratory of Cancer Prevention and Therapy of Tianjin, Tianjin Clinical Research Center for Cancer, Tianjin, China; ^4^ The Pelotonia Institute for Immuno-Oncology at The Ohio State University Comprehensive Cancer Center, Columbus, OH, United States

**Keywords:** endoplasmic reticulum stress, cancer, chaperone, GRP94/GP96, biomarker, therapeutic target

## Abstract

During tumor development and progression, intrinsic and extrinsic factors trigger endoplasmic reticulum (ER) stress and the unfolded protein response, resulting in the increased expression of molecular chaperones to cope with the stress and maintain tumor cell survival. Heat shock protein (HSP) GRP94, also known as GP96, is an ER paralog of HSP90 and has been shown to promote survival signaling during tumor-induced stress and modulate the immune response through its multiple clients, including TLRs, integrins, LRP6, GARP, IGF, and HER2. Clinically, elevated expression of GRP94 correlates with an aggressive phenotype and poor clinical outcome in a variety of cancers. Thus, GRP94 is a potential molecular marker and therapeutic target in malignancies. In this review, we will undergo deep molecular profiling of GRP94 in tumor development and summarize the individual roles of GRP94 in common cancers, including breast cancer, colon cancer, lung cancer, liver cancer, multiple myeloma, and others. Finally, we will briefly review the therapeutic potential of selectively targeting GRP94 for the treatment of cancers.

## Introduction

**Figure 1:**
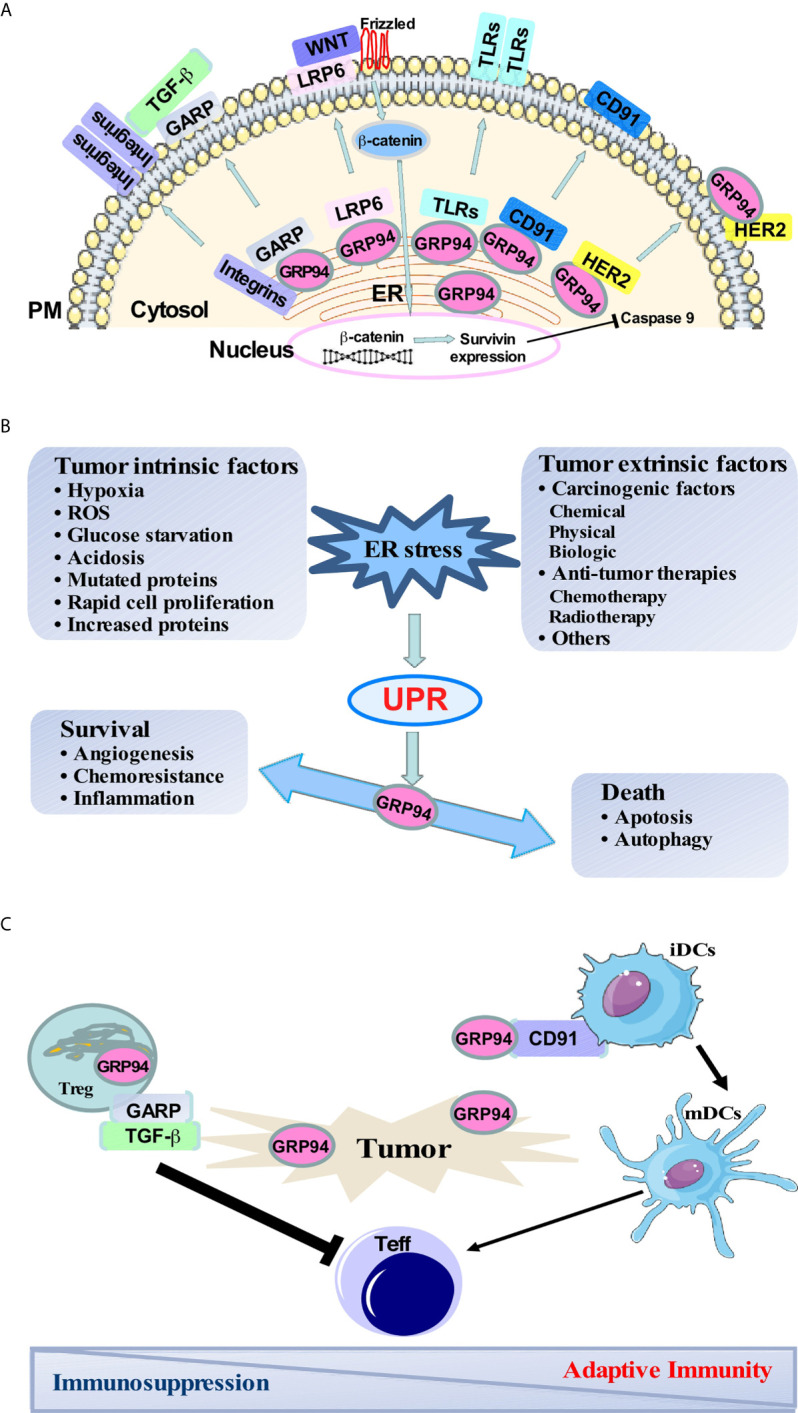
GRP94 major client network, the role of GRP94 in balancing cancer-induced ER stress responses, and immune regulation in the tumor microenvironment. **(A)** Major clients of GRP94 and its role in different signaling pathways. **(B)** Both intrinsic and extrinsic stress conditions exist in the tumor microenvironment and trigger ER stress and UPR. Activation of UPR leads to the up-regulation of pro-survival signaling involved in angiogenesis, folding capacity, redox protection, and degradation of unfolded proteins to keep the cell surviving. However, when the UPR is prolonged, it can also result in cell death through apoptosis and autophagy. GRP94 is a downstream molecule of UPR. The increase of GRP94 is responsible for strengthening pro-survival signaling to promote tumor cell survival and aggression. **(C)** Surface GRP94 is involved in DC maturation *via* a cell surface receptor CD91. Peptides chaperoned by GRP94 can be presented to cytotoxic T lymphocytes and initiate antigen-specific T-cell responses to cancer. Also, GARP is translocated to the cell surface with the help of GRP94, which then activates latent TGF-β in regulatory T cell (Tregs). Tregs then mediate the suppression of effector T cells leading to immunosuppression.

Glucose regulated protein 94 (GRP94), also known as GP96, is a stress-inducible molecular chaperone that belongs to the heat shock protein (HSP) 90 family (1). GRP94 is upregulated in many stress conditions that disturb endoplasmic reticulum (ER) homeostasis (2). A wide range of stressful conditions exists within the tumor microenvironment, including hypoxia, redox homeostasis dysregulation, altered cell metabolism, acidosis, and genetic lesions leading to the production of mutated proteins, high rates of proliferation, and increased protein synthesis (3). Activation of the unfolded protein response (UPR) leads to the up-regulation of pro-survival proteins involved in angiogenesis, folding capacity, redox protection, or degradation of unfolded proteins (4). However, when the activation of this response is prolonged, it can also result in cell death. GRP94 plays a critical role in regulating the balance between cancer cell survival and death through sustaining ER protein folding capacity, maintaining ER stress sensors, and repressing ER-associated pro-apoptotic machinery (**Figure 1B**).

Moreover, GRP94 is responsible for chaperoning multiple proteins that have been reported to play essential roles in immune response and promoting cancer development, including Toll-like receptors (TLRs) (5–8), the majority of α and β integrin subunits (7, 9), Wnt co-receptor low-density lipoprotein receptor-related protein 6 (LRP6) (9, 10), glycoprotein A repetitions predominant (GARP) (11–15), Insulin-like growth factor (IGF) (16–18), as well as platelet glycoprotein Ib-IX-V complex (19) (**Figure 1A**).

Using a genetic strategy, depletion of GRP94 from specific cells reveals that GRP94 promotes tumorigenesis in multiple myeloma (10), liver cancer (20–22), colon cancer (23, 24), and endometrial carcinoma (25). An elevated level of GRP94 has also been reported in many cancers (26–29) and is associated with a more aggressive tumor phenotype. This review will mainly focus on the involvement of GRP94 in cancer development and progression and its potential as a diagnostic biomarker as well as a therapeutic target.

## Oncogenic Nature of GRP94 CLIENTS

GRP94 is a master ER chaperone that plays a role in protein quality control in response to stress, inflammation, and cancer through its client protein networks, such as LRP6-Wnt, GARP-TGF-β signaling pathways, etc. (9, 10, 23). The Wnt signal network plays a critical role in regulating cell differentiation, proliferation, and fate (30, 31). The bindings of Wnt to Frizzled and LRP 5 and 6 stabilize β-catenin, a major mediator in the canonical Wnt signaling pathway (32, 33). GRP94 is a critical chaperone for the Wnt co-receptor LRP6. Without GRP94, LRP6 fails to export from the ER to the cell surface, resulting in a profound loss of canonical Wnt signaling and causing a fast and severe compromise in intestinal homeostasis. This study uncovered the role of GRP94 in coordinating intestinal homeostasis through chaperoning LRP6, placing the canonical Wnt-signaling pathway under the direct regulation of the general protein quality control machinery in the ER (9). Additionally, specific deletion of GRP94 in macrophages protects mice from inflammation-associated colon tumorigenesis partially by the protection of gut epithelium from β-catenin mutation and stabilizes the DNA repair pathway (23). Furthermore, we demonstrated that GRP94 is required for multiple myeloma cell survival, which is mediated in part by the Wnt target survivin in a murine model of multiple myeloma (10). Also, we found that GRP94 is highly expressed in malignant plasma cells in human multiple myeloma, and the higher levels of GRP94 have a significant association with a worse clinical stage in myeloma (34).

GARP is essential to the expression of latent TGF-β (LTGF-β) on the surface of Foxp3^+^ regulatory T cells (Tregs) and activated platelets (35). Zhang et al. demonstrated that GARP is a client protein of GRP94 (11). In the absence of GRP94, GARP is unable to exit the ER because of incorrect protein folding. Specific deletion of GRP94 in Tregs resulted in both membrane-associated LTGF-β and secreted form of active TGF-β from Tregs being significantly reduced (11) (**Figure 1C**). Also, platelet-specific deletion of GARP blunted systemic active TGF-β and induced anti-tumor immunity against both melanoma and colon cancer (13). Furthermore, Salem et al. demonstrated that Treg cells lacking GARP were unable to suppress pathogenic T cell responses, promoted inflammation, and improved anti-tumor immunity in the inflammation-associated colon cancer model (14). Moreover, Metelli et al. found that GARP is highly expressed in human breast cancers compared with normal breast tissue. Overexpression of GARP in normal mammary gland epithelial cells increased TGF-β bioactivity and promoted malignant transformation in immune-deficient mice (12).

GRP94 is a master chaperone for a variety of integrins and all TLRs except TLR3 (5–9, 36, 37). Integrins are transmembrane receptors that facilitate cell-extracellular matrix interaction and signal transduction by modulating the cell signaling pathways of transmembrane protein kinases that can promote cell transformation and tumor progression (38, 39). TLRs are a class of proteins that recognize the pathogen and damage associated-molecular patterns and play a key role in innate and adaptive immune responses. TLRs are localized either on cell surfaces (TLR1, 2, 4, 5, and 6) or in the endosomes (TLR 3, 7, 8, and 9). Surface TLRs are responsible for primarily recognizing bacteria components. Intracellular TLRs recognize nucleic acids such as TLR3 for dsRNA, TLR7/8 for ssRNA, and TLR9 is the receptor for unmethylated DNA enriched with the CpG motif (40, 41). Both immune cells and tumor cells express TLRs. Immune cells of the myeloid and lymphoid lineages express TLRs to recognize pathogenic components or cellular debris and activate the immune system through the secretion of cytokines and chemokines, thereby recruiting immune cells into the tumor microenvironment and playing a key role in innate and adaptive immune responses (42).

Accumulating evidence demonstrates that the TLR signaling can be a double-edged sword in the tumor microenvironment (42–44). TLRs can manifest either pro-or anti-tumor activities depending on the tumor-infiltrating immune cells and cancer type. However, controversies exist regarding some TLRs in experimental tumor models (45). The TLR stimulation in an experimental tumor model has an anti-tumor effect by directing immune cells to a tumor site or reducing tumor progression by enhancing tumor cell apoptosis. On the contrary, TLRs expressed by tumor cells have been widely associated with tumor progression. TLR2 plays both anti- and pro-tumor roles depending on its expression on immune cells or tumors. Lowe et al. showed that TLR2^−/−^ mice developed more tumors in a colitis-associated colorectal tumor mouse model (46). However, specific deletion of TLR2 in epithelial cells protects APC^min/+^ mice from adenomas and delayed the onset of mammary tumor development (47). A recent study also showed that the tumor expressing high level of TLR2 and TLR4 in colorectal cancer patients was associated with a worse disease (48). Another study showed that stimulation of TLR2 promotes TLR2 positive squamous carcinoma cell growth (49). However, Reilley et al. showed that local injection of TLR9 agonist improved checkpoint blockade immunotherapies in poorly immunogenic melanoma (50). Also, Bellmann et al. reported that a clinical approved TLR7 agonist increased activation of tumor-infiltrating T and NK cells, and delayed melanoma cells’ resistance to BRAF inhibitor (51). Thus, care must be taken while designing anti-cancer drugs targeting TLRs due to their dual role.

Human epidermal growth factor receptor-2 (HER2) is a receptor tyrosine kinase (RTK). Overexpression/amplification of HER2 has been shown in different cancers and is associated with worse clinical outcomes. HER2-targeted therapies have been used in the treatment of HER2-positive breast cancer, gynecologic malignancies, and other cancers (52–54). Interestingly, Chavany et al. demonstrated that GRP94 modulates HER2 intracellular trafficking and stability (55). Later on, Patel et al. discovered that GRP94 is associated with HER2 and stabilizes this protein at the plasma membrane (PM) in the HER2-positive breast cancer cells (56). Li et al. further demonstrated that GRP94 interacts with HER2 and facilities HER2 dimerization on the PM. Overexpression of GRP94 on the PM promotes the growth of HER2-positive breast cancer. Targeting GRP94 with a monoclonal antibody inhibits tumor growth (57). These studies indicate that molecule chaperone GRP94 and its clients play a pivotal role in cancer initiation and development.

## Surface Expression of GRP94 and Tumor Immunogenicity

GRP94/GP96 normally resides in the lumen of the ER. However, it can be translocated to the cell surface under stress and other conditions such as bacterial infection (58, 59) and in the tumor microenvironment (60, 61). GRP94 is a peptide-binding protein and stimulate an anti-tumor immune response (62). The selective surface expression of GRP94 on some immune cells in different vertebrate classes is consistent with an ancestral immunological role of GRP94 as a danger-signaling molecule (61).

Dendritic cells (DCs) are professional antigen-presenting cells (APCs) and are central to the regulation, maturation, and maintenance of cellular immune response to cancer. After taking up antigens, immature DCs differentiate into mature DCs that prime naive T cells and initiate antigen-specific T-cell responses to tumors (63). Zheng et al. discovered that surface expression of GRP94 on tumor cells is involved in DC maturation and activation as well as increases tumor immunogenicity to suppress tumor growth through T lymphocytes (64). Furthermore, cell surface expression of GRP94 enhances cross-presentation of cellular antigens and the generation of tumor-specific T cell memory (65). Peptides chaperoned by GRP94 can be presented to cytotoxic T lymphocytes. Such a presentation requires the uptake of GRP94 *via* a cell surface receptor CD91 expressed by DCs (64) (**Figure 1C**).

On the other hand, cell surface GRP94 has also been found on cancer cells. A recent study found that HER2^+^ breast cancer cells expressed GRP94 on the PM, and GRP94 maintained the stability of HER2 to enhance downstream signaling (56). More recently, Yan et al. found that different cancer cells had distinct sensitivities to GRP94 inhibitors. More GRP94 was located on the PM in inhibitor-sensitive breast cancer cells compared with inhibitor-insensitive cancer cells. Also, the GRP94 inhibitor is preferentially bound to the PM-localized GRP94-HER2 complex. Interestingly, the hyper-N-glycosylated GRP94 at the PM contributed to the stability of GRP94-receptor tyrosine kinase complexes, which indicates that GRP94 can adopt a unique hyperglycosylated conformation to preferentially regulate growth factor receptors on tumor cells and alter tumor oncogenesis (66) (**Figure 1A**).

## The Ontogenetic Role of GRP94 in Cancers

### Multiple Myeloma

Multiple myeloma (MM) is a plasma cell malignancy. UPR plays a critical role in plasma cell differentiation and myeloma pathogenesis (67, 68). UPR is an evolutionally conserved mechanism that maintains protein quality control in the secretory pathway. Accumulation of misfolded proteins in the ER triggers the activation of three well-known pathways: activating transcription factor 6 (ATF6), the double-stranded RNA-activated protein kinase-like ER kinase (PERK), and the spliced form of X-box binding protein 1 (XBP1s). XBP1s and downstream ER chaperones are consistently upregulated in myeloma cells (69). Malignant plasma cells characteristically produce large amounts of proteins in the form of immunoglobulins that require ER chaperones such as GRP94 to prevent ER stress-induced cell death. Hua et al. found that the persistence of plasma cells and the development of myeloma in XBP1s-transgenic mice are critically dependent on GRP94. The addiction of myeloma cells to GRP94 was also demonstrated genetically and pharmacologically using multiple human myeloma cell lines (10). Mechanically, GRP94 is a critical chaperone for LRP6 and is essential for canonical Wnt signaling. LRP6 is required for the release of β-catenin from its destruction complex, accumulation of nuclear β-catenin, and upregulation of Wnt targets including survivin. Deletion of GRP94 compromises survivin expression, leading to its failure to safeguard mitotic spindles and the initiation of apoptosis (10, 70) through the activation of proapoptotic molecule CHOP, which causes downstream activation of the JNK and intrinsic caspase pathways that finally lead to apoptosis of tumor cells (71). Also, Chhabra et al. found that GRP94 is highly expressed in malignant plasma cells in MM. The higher level of GRP94 is significantly associated with a worse clinical stage in MM (34). These studies uncover the critical roles of GRP94 in the initiation and progression of MM, suggesting that blockade of GRP94 is a novel therapeutic strategy against this disease.

### Breast Cancer

An early study showed that GRP94 is highly expressed in breast carcinoma cells but not in normal mammary tissue (26). CD44^hi^/CD24^lo^ breast cancer stem cells represent the main driving factor in breast cancer initiation, growth, metastasis, and poor responses to anti-cancer agents. Nami et al. found that CD44^hi^/CD24^lo^ cells exhibited higher expression of GRP94 at both mRNA and protein levels compared to their original bulk cells (72). Dejeans et al. found that overexpression of GRP94 in breast cancers is associated with resistance to oxidative stress and the promotion of cancer cell proliferation and migration (4). The expression level of GRP94 was higher in recurrent human breast cancers than in their paired primary tumor (29). Recent studies also showed that GRP94 overexpression is associated with brain metastasis and poor survival in breast cancer patients (73, 74). Upregulation of the GRP94 is associated with triple-negative breast cancer brain metastasis through the Wnt-β-catenin signaling pathway (75). A recent study showed that GRP94 promoted brain metastasis by engaging pro-survival autophagy (76). Patients with infiltrated axillary lymph nodes also displayed increased expression of GRP94 protein.

Recently, Hou et al. found that GRP94 also regulated ER-α36 expression and signaling on the cell membrane of breast cancer. ER-α36 is a variant of human ERα that is overexpressed in breast cancer and involved in tamoxifen resistance. Targeting GRP94 with siRNA or monoclonal antibody blocked the GRP94-ER-α36 interaction and inhibited breast cancer growth and invasion (57). Also, GRP94 can control the stability of the nascent and mature forms of HER2, an RTK, which leads to the upregulation of numerous cancer-driving signaling pathways (77). GRP94 specific inhibitors provide evidence for the role of GRP94 in maintaining the architecture of high-density HER2 formations at the plasma membrane, which is vital for proper HER2 functioning in breast cancers (56). Targeting GRP94 with a specific monoclonal antibody or peptide-based inhibitor p37 disrupted HER2 dimerization and led to HER2 degradation, which subsequently decreased tumor cell growth and increased apoptosis (57, 78). These results provide insights into the dependence of breast cancer cells on GRP94 for survival and suggest that GRP94 could be a potential therapeutic target for HER2 or ER-α36-overexpressing breast cancer.

### Colon Cancer

The intestinal epithelial cells are continually replenished through the proliferation and differentiation of intestinal stem cells within the intestinal crypts (79). The interplay between ER stress and inflammation contributes to the pathogenesis of inflammatory bowel diseases and inflammation-associated colon cancer (80). Canonical Wnt signaling through the surface receptor Frizzled and its coreceptors LRP5 or LRP6 is essential for the homeostatic proliferation of epithelial cells in the gut. Unsurprisingly aberrant Wnt/β-catenin signaling also plays a role in the initiation and progression of colorectal cancer, as well as its invasion and metastasis (81). Epithelial-specific GRP94 deficiency resulted in the loss of intestinal barrier function in mice dependent on canonical LRP6/Wnt-signaling (9). This study uncovered the role of GRP94 in chaperoning LRP6-MesD to coordinate intestinal homeostasis, placing the canonical Wnt-signaling pathway under the direct regulation of the general protein quality control machinery in the ER (9).

Macrophages are important drivers in the development of inflammation-associated colon cancers. Using a unique macrophage-specific GRP94 deficient mouse model, Morales et al. found that macrophages promoted colitis and colitis-associated colon tumorigenesis in a GRP94-dependent manner. Strikingly, they also discovered that deletion of GRP94 in macrophages attenuated colon tumor initiation, which was correlated with reduced mutation rates of β-catenin, reduced activation of the canonical Wnt signaling, and increased efficiency of the DNA repair machinery as well as reduced expression of pro-inflammatory cytokines, including IL-17 and IL-23 in the tumor microenvironment (23). These studies reveal that GRP94 is a strategically important ER chaperone that integrates stress and innate immunity and plays a pivotal role in macrophage biology and tumor oncogenesis. Besides, GRP94 is upregulated in human metastatic colorectal cancer (28). Therefore, targeting GRP94 in macrophages may prove to be an attractive strategy for the treatment of colon cancer by regulating the Wnt and inflammatory signaling in the tumor microenvironment.

### Liver Cancer

The liver is an exocrine and endocrine organ involved in the synthesis of bioactive molecules and proteins, detoxification, and metabolism, resulting in a particularly high need for adequate ER maintenance and stress-coping mechanisms. Hence, the loss of GRP94 in hepatocytes would drastically affect normal physiological functions. Chen et al. demonstrated that conditional deletion of GRP94 and PTEN from the mouse liver increased liver tumorigenesis (20). However, the GRP94 status of these tumors was not reported, leaving open a possibility of GRP94 being either a tumor suppressor or a pro-oncogenic chaperone (21). Using a similar strategy, Rachidi et al. (22) found that GRP94 maintains liver development and hepatocyte function *in vivo*. Deletion of GRP94 in hepatocytes promotes adaptive accumulation of long-chain ceramides, accompanied by steatosis and regeneration of residual GRP94^+^ hepatocytes. The need for compensatory expansion of GRP94^+^ cells in the GRP94^-^ background predisposes mice to develop carcinogen-induced hepatic hyperplasia and cancer from GRP94^+^ but not GRP94^-^ hepatocytes. They also demonstrated that both genetic and pharmacological inhibitions of GRP94 in human hepatocellular carcinoma cells perturbed multiple growth signals and attenuated their proliferation and expansion. The development of GRP94^+^ but not GRP94^-^ tumors in the same hosts indicated that GRP94 played tumor-promoting rather than a tumor-suppressive role in liver cancer (21). Similar results were found in another study. Deletion of GRP94 resulted in liver injury, activation of oncogenic signaling, repopulation of GRP94^+^ hepatocytes, and spontaneous development of hepatocellular carcinoma in aged mice (82).

Additionally, Lim et al. showed that the expression of GRP94 is up-regulated in hepatitis B virus-related hepatocellular carcinoma in humans, and is strongly correlated with vascular invasion and intrahepatic metastasis (83). Wei et al. also found that knockdown of GRP94 inhibited the growth, invasion, and metastasis of hepatocellular carcinoma cells *in vivo* and *in vitro*. The high expression of GRP94 in tumors indicated poor survival (84, 85). These studies show the oncologenic role of GRP94 and its potential as a prognostic indicator of liver cancer.

### Lung Cancer

Cigarette smoking is the most relevant environmental risk factor associated with chronic obstructive pulmonary disease (COPD) and lung malignancies. It is well established that cigarette smoke induces ER stress, which activates UPR, and COPD subjects have intense ER stress evidenced by high expression of ER stress markers in fully differentiated human bronchial epithelial cells (86). Chronic ER stress upon exposure to cigarette smoking or other causative agents may play a pivotal role in the etiology or progression of lung cancers (87). UPR activation and enhanced ER chaperon translation, including GRP94 may promote lung cancer progression.

Wang et al. revealed that GRP94 is overexpressed in lung cancer at the mRNA and protein level and correlated with poor epithelial differentiation and tumor progression (88). Lee et al. showed that 98% of small cell lung cancer patients were GRP94 positive with moderate to high expression (27). GRP94 was also highly expressed in non-small cell lung cancer patients with brain metastasis (74). Furthermore, the upregulation of GRP94 by the interference of calcium stores can confer drug resistance to lung cancer cells against the chemotherapy agent etoposide (89). A recent study demonstrated that GRP94 is highly expressed in lung adenocarcinoma and is associated with an advanced stage of the disease as well as poor survival. Also, the GRP94 expression level was positively correlated with FoxP3^+^ regulatory T cells in tumors (90). Hence, targeting GRP94 will provide a new therapeutic approach to the clinical management of lung cancer with chemo-resistance.

### Others

Elevated expression of GRP94 correlates with the aggressiveness of numerous other tumors, including esophageal (91), gastric (92), and pancreatic cancers (93) as well as oral carcinoma (94) and glioblastoma (95), indicating that GRP94 plays a tumor-promoting role in many different cancers. Shen et al. generated a uterus-specific GRP94 knockout mouse model and discovered that GRP94 suppressed PTEN-null driven endometrioid adenocarcinoma. Deletion of GRP94 reduced cellular proliferation through attenuating β-catenin signaling and decreasing AKT/S6 activation (25).

Tramentozzi et al. found that GRP94 was highly expressed in both gastrointestinal tumor tissues and tumor-infiltrating lymphocytes regardless of stage or anatomical location. GRP94 was also found in the plasma in stable complexes with Immunoglobulin G (IgG). The study showed that GRP94-IgG complexes are significantly increased in cancer patients compared to healthy control subjects, suggesting its potential as a diagnostic biomarker. GRP94 is over-expressed in a wide variety of both solid and hematological tumors and correlates with poor outcomes, which indicates that GRP94 is a potential diagnostic and prognostic biomarker.

## GRP94/GP96 and Cancer Therapeutics

### Tumor Vaccine

Mounting evidence indicates that the surface expression of GRP94/GP96 plays an integral role in shaping the immune landscape of tumors. Melendez et al. found that surface expression of GRP94 in malignant breast cells correlates with NK-mediated cytotoxicity, and the use of a GRP94 blocking antibody protected tumor cells from NK cytotoxicity (96). Zheng et al. found that overexpressed surface GRP94 on colon cancer and fibrosarcoma cells are capable of inducing maturation of dendritic cells leading to the release of proinflammatory cytokines and upregulation of antigen presentation machinery (64). Similarly, another study found that the administration of a GRP94 and Her2/neu DNA vaccine to HER2^+^ breast cancer-bearing mice led to an increased immune response against the tumors evidenced by increased IFN-γ/IL-4 levels and decreased Tregs at the tumor site (97). These studies reveal the ability of extra-cellular GRP94 to induce anti-tumor immune responses, and highlighting its potential to be used as an antigen for tumor vaccines (**Table 1**).

**Table 1:** GRP94/GP96-targeted treatment strategies for cancers.

Strategies	Phase	Effect	Reference
**Tumor Vaccine**
Surface GP96-tumor cells	Pre-clinical study	Suppressed tumor growth through T cell-dependent mechanism.	(64)
GRP94/Her2/neu DNA vaccine	Pre-clinical study	Decreased Tregs at the tumor site, increasing IFN-γ/IL-4 level; partial control of tumor progression.	(97)
Placental-derived GRP94	Pre-clinical study	Exhibited high immunogenicity against multiple tumors (melanoma, HER^+^, and triple negative mammary tumors).	(98)
Tumor-derived GP96	I/II	Increased tumor-specific immune response and prolonged the survival of patients with metastatic CRC and gastric cancer.	(99, 100)
GP96-Ig vaccine	Pre-clinical study	Increased Ag-specific CD8^+^ and memory T cells, delayed melanoma tumor growth and prolonged overall survival.	(101)
DC vaccine with tumor-derived GP96	Pre-clinical study	Decreased the tumor growth of murine lung cancer through CD8^+^ T cells and NK cells.	(102)
**Small molecule inhibitor**			
PU-WS13	Pre-clinical study	Induced apoptosis of HER2^+^ breast cancer cells.	(56)
PU-WS13	Pre-clinical study	Induced apoptosis and inhibited the growth of multiple myeloma through Wnt-Survivin pathway.	(10)
PU-WS13	Pre-clinical study	Inhibited the proliferation of hepatocellular carcinoma.	(22)
GRP94 Inhibitor 30	Pre-clinical study	Reduced the migratory capabilities of metastatic breast and prostate cancer cells through degradation of integrin α2.	(103)
**Monoclonal antibody**			
Anti-GP96 mAb	Pre-clinical study	Increased apoptosis and suppressed the HER2^+^ breast cancer cell growth.	(57, 78)
Anti-GRP94 mAb W9	Pre-clinical study	Selectively recognized GRP94-epitope on malignant cells. Increased and restored the sensitivity of BRAF-1-resistant melanoma Cells.	(104)
Anti-sGRP94 IgG	Pre-clinical study	Inhibited the growth of Cetuximab-resistant CRC cells.	(105)

Immunization of tumor-bearing mice with GRP94-peptide complexes has been shown to generate effective immune responses in numerous pre-clinical and clinical settings (99, 106). Autologous tumor-derived GRP94/GP96 significantly attenuated tumor growth and improved survival in various spontaneous and carcinogen-induced cancer models. The effectiveness of the treatment was dependent on the presence of CD4^+^ and CD8^+^ T cells and NK cells (106). Similarly, placental derived GRP94 was found to bind to tumor-associated antigens such as HER2 and MUC1 and induce tumor-specific T cell responses (98). The therapeutic effectiveness of GRP94 complexes likely results from their ability to improve the uptake and processing of tumor antigens. Consequently, Shinagawa et al. found that delivering GRP94 complexes directly to dendritic cells *ex vivo* before adoptive transfer of DCs into tumor-bearing mice led to improved tumor control compared to treatment with DCs or tumor-derived GRP94 complexes alone (102). Fromm et al. reported that the secreted form of GRP94 (GRP94-Ig) and costimulator combination cellular vaccine increased antigen-specific CD8 and memory T cells, delayed melanoma tumor growth, and prolonged overall survival (101) (**Table 1**).

### Small Molecular Inhibitors

GRP94 is expressed in most human cells. Therefore, specificity for cancer cells is a major concern when designing small molecular inhibitors. Identification of geldanamycin (GM) revealed the possibility of selectively targeting HSP90 within tumor cells. A more recent study showed that the purine scaffold small molecule DN401 simultaneously inhibited all HSP90 paralogs, including HSP90, TRAP1, and GRP94, and synergized the anti-tumor effects with another TRAP1 inhibitor, gamitrnib (107). However, pan HSP90 inhibitors inactivate all HSP90 isoforms, including GRP94, and display severe toxicity. To improve selectivity and further reduce toxicity, Patel et al. developed novel purine-based ligands that are greater than 100-fold more selective for GRP94 over HSP90α/β (56). Based on a strategy that combined library screening of purine-scaffold compounds and structural studies, GRP94-specific inhibitors, PU-WS13, PU-H39, and PU-H54, were developed, and these inhibitors significantly induced apoptosis of human HER2 positive breast cancer cells (56). Also, Hua et al. found that PU-WS13 significantly inhibited the growth of multiple human MM cells, including those that are resistant to cytosolic HSP90 inhibitor PU-H71, doxorubicin, and proteasome inhibitor bortezomib *in vitro* (10). Recently, based on an extensive Structure–Activity relationship study, Patel et al. designed a new GRP94-specific inhibitor, compound 18c. Specifically, 18c inhibited the post-ER expression of TLR9, and also reduced the steady-state levels of HER2 kinase in HER2-overexpressing breast cancer cells. When administered into tumor-bearing mice, compound 18c was cleared rapidly from normal tissue while being selectively retained in the tumor (108). Crowley et al. developed another series of GRP94-selective inhibitors (103). These small molecular inhibitors reduced the migratory capabilities of metastatic breast and prostate cancer cells through the degradation of integrin α2 and with better selectivity than their previous reported compounds (109–112) (**Table 1**). Recently, the Xu group developed a GRP94 selective inhibitor, Compound 54, with an IC50 of 2 nM and over 1,000-fold selectivity for GRP94 versus HSP90α. They found that this compound has anti-inflammation efficacy in the DSS-induced colitis mouse model (113). However, Compound 54 hasn’t been tested in the treatment of cancers.

### Monoclonal Antibody

The therapeutic targeting of surface GRP94 in tumors has emerged with the recent development of specific monoclonal antibodies that have potent GRP94 inhibitory effects and consequent anti-tumor activities (114). Li et al. developed a GRP94 monoclonal antibody that interfered with GRP94-dependent HER2 dimerization and phosphorylation in breast cancer and suppressed the HER2^+^ breast cancer cell growth *in vitro* and *in vivo* (57, 78). Moreover, Sabbatino et al. developed a novel grp94-antagonizing monoclonal antibody, W9 mAb, which selectively targets the extracellular epitope of GRP94 in malignant cells but is not detectable on normal cells. W9 mAb increased the sensitivity of human BRAF^V600E^ melanoma cells to BRAF inhibitors (104). Using a phage display approach, Jeoung et al. isolated an antibody binding to the cell surface of human colon cancer cells. Furthermore, they demonstrated that this antibody specifically targeted GRP94 and inhibited the growth of Cetuximab-resistant colorectal cancer (105) (**Table 1**). These studies lay a strong foundation for developing GRP94-targeted therapeutics for cancers in the future.

## Conclusion

A wide range of intrinsic and extrinsic stressful conditions exists within tumors during development and progression, creating the need for increased chaperone expression to cope with the stress and regulate the balance between tumor cell viability and death (**Figure 1B**). The elevated expression of GRP94 in a variety of cancer cells correlates with an aggressive phenotype, giving it strong potential as a biomarker and therapeutic target. GRP94 has been revealed to play roles in survival signaling through its client protein network, induction of the UPR, and modulating the immune response (**Figure 1**). The development of a GRP94-based tumor vaccine, small molecular inhibitors, and monoclonal antibodies will open new territories for cancer treatment. Nevertheless, previous attempts to target HSP90 have not been successful in clinical trials due to severe toxicity. Recently, Yan et al. discovered that GRP94 adopt a unique hyperglycosylated conformation to preferentially regulate growth factor receptors on tumor cells. GRP94 specific inhibitor PU-WS13 favorably binds to hyperglycosylated GRP94 and inhibits RTK-driven tumor growth (66). Selective inhibition of GRP94 is still a feasible targeted therapy. Up to date, the complete GRP94 client network is still unknown. Identification of GRP94 clients involved in tumor initiation and progression and the development of specific GRP94 client-targeted therapeutics will bear fruit for the treatment of cancers.

## Author Contributions

Conceptualization, BL. Writing—original draft and literature review, XD and BL. Writing—review and editing, XD, SI, SN, MH, QZ, and BL. All authors contributed to the article and approved the submitted version.

## Funding 

This work was supported in part by NIH NCI (T32: CA193201), NIH NIAID (T32: AI 132164), NIH NCI (R01: CA193939), and NIH NIAID (U01: AI125859).

## Conflict of Interest

The authors declare that the research was conducted in the absence of any commercial or financial relationships that could be construed as a potential conflict of interest.
